# Supplementary material for: A biochemical network controlling basal myosin oscillation
Source: Nat Commun. 2018 Mar 23;9:1210. doi: 10.1038/s41467-018-03574-5 (PMC5865161; doi:10.1038/s41467-018-03574-5)
Supplement: Supplementary file 1 — Supplementary Information(PDF 3539 kb) [file 41467_2018_3574_MOESM1_ESM.pdf]

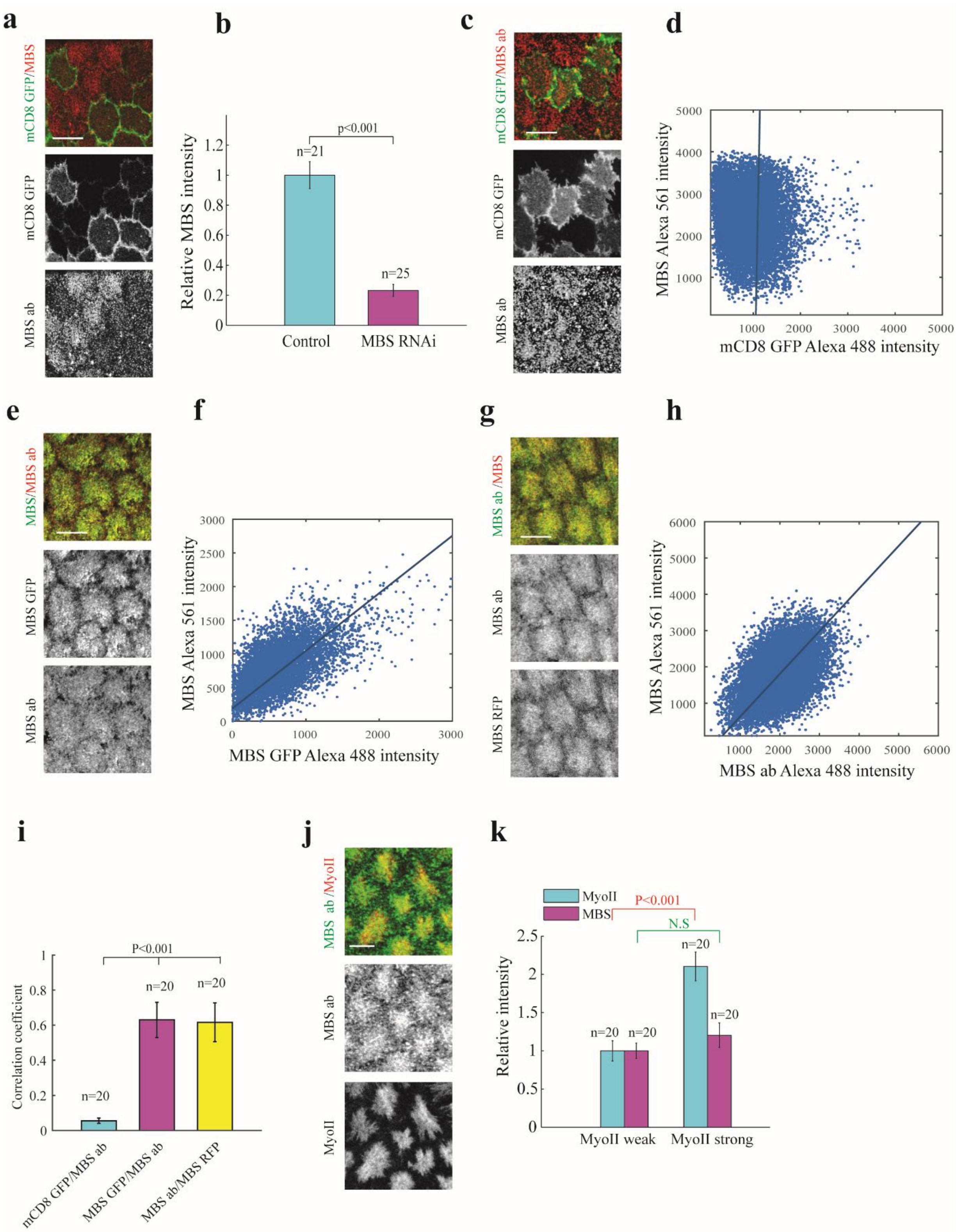

Supplementary Figure 1. Confirmation of the MBS knockdown efficiency, the endogenous MBS levels correlated with Myo-II signals, and with MBS reporters

a. Confocal micrographs of endogenous MBS signals, monitored by MBS antibody staining, in follicle cell clones expressing MBS RNAi, marked by coexpression of mCD8GFP. **b.** Quantification of relative MBS intensity in the MBS RNAi-expressing GFP-positive cells

compared with wild type cells in the same tissue sample. **c, e, g.** Confocal micrographs of follicle cells expressing mCD8-GFP (**c**), MBS-GFP (**e**), or MBS-RFP (**g**), stained with MBS antibody. **d, f, h.** Similarity comparisons between mCD8-GFP (**d**), MBS-GFP (**f**), or MBS-RFP (**h**) and endogenous MBS signals, monitored by MBS antibody staining. **i.** Average spatial cross-correlation between the indicated signals. **j.** Basal view of follicle cells with the presence of MyoII-mCherry, stained with MBS antibody. All scale bars are 10 μm. **k.** Quantification of relative Myo-II and MBS intensities from follicle cells with strong vs. weak Myo-II signals in the same tissue sample. n is the number of samples analyzed. Error bars indicate ±s.d. N.S means no significant difference, while $p<0.001$ means significant difference by student's *t*-test.

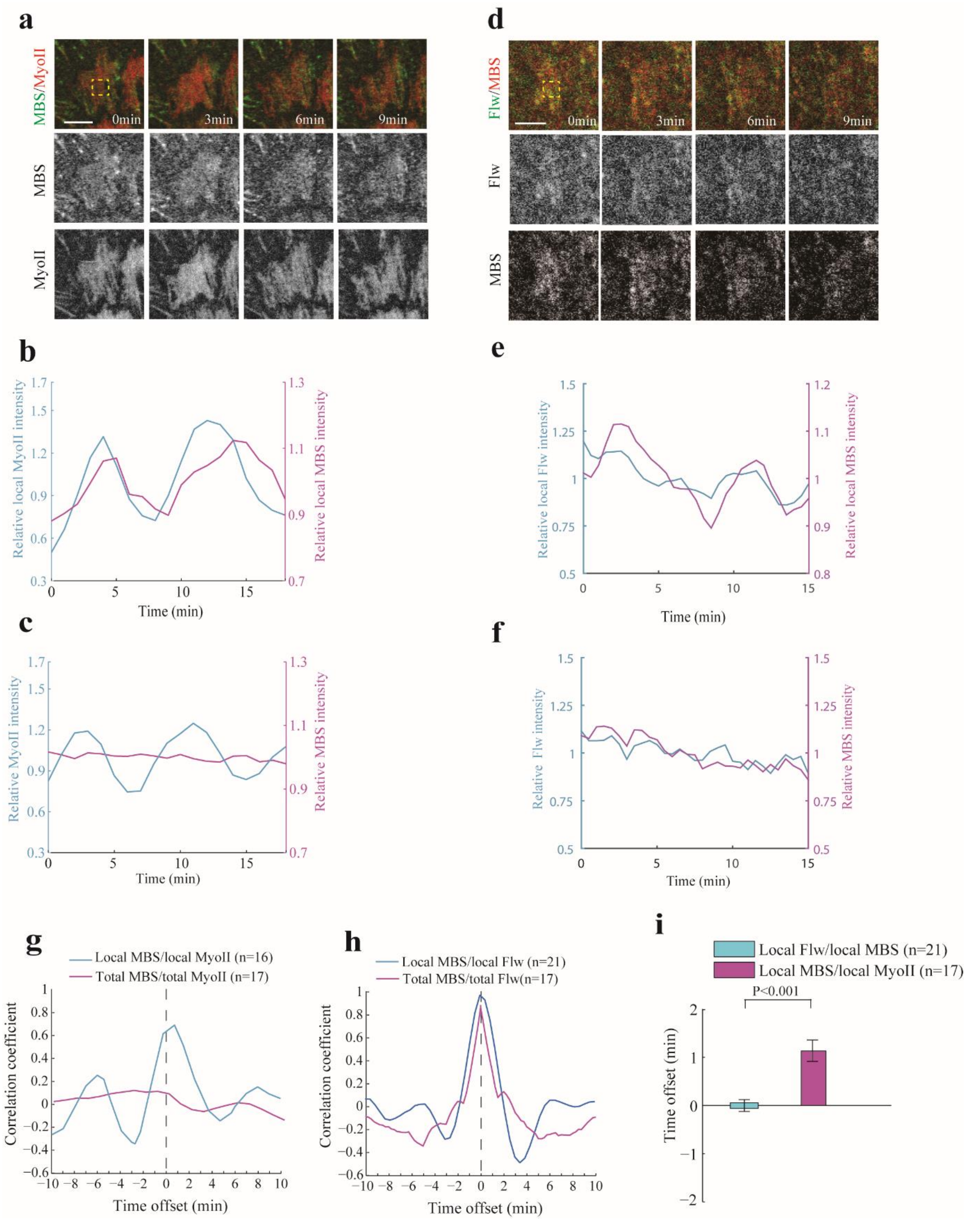

Supplementary Figure 2. MBS pulses are synchronized with Flw at the medial basal cortex

a, d. Time-lapse series of the representative oscillating follicle cells, labelled with MBS-GFP and MyoII-mCherry (**a**) or Flw-YFP and MBS-mCherry (**d**), respectively. Both scale bars are 5 μm. **b, c, e, f.** Quantifications of the dynamic change of MBS-GFP and MyoII-mCherry intensities (**b, c**) and Flw-YFP and MBS-mCherry intensity (**e, f**) in a local medial basal region of one oscillating cell (**b, e**) or in one oscillating cells (**c, f**). A local medial basal region

has been marked by a dotted square. Intensity of each channel is normalized to its mean. **g, h.** Average temporal cross-correlation of MyoII-mCherry with local MBS-GFP (cyan) or total MBS-GFP (magenta) (**g**), local MBS-mCherry with local Flw-YFP (cyan), total MBS-mCherry with total Flw-YFP (magenta) (**h**). **i.** Time offset calculated from the cross-correlation analysis. n is the number of samples analyzed. All error bars indicate ±s.d. $p<0.001$ means significant difference by student's *t*-test.

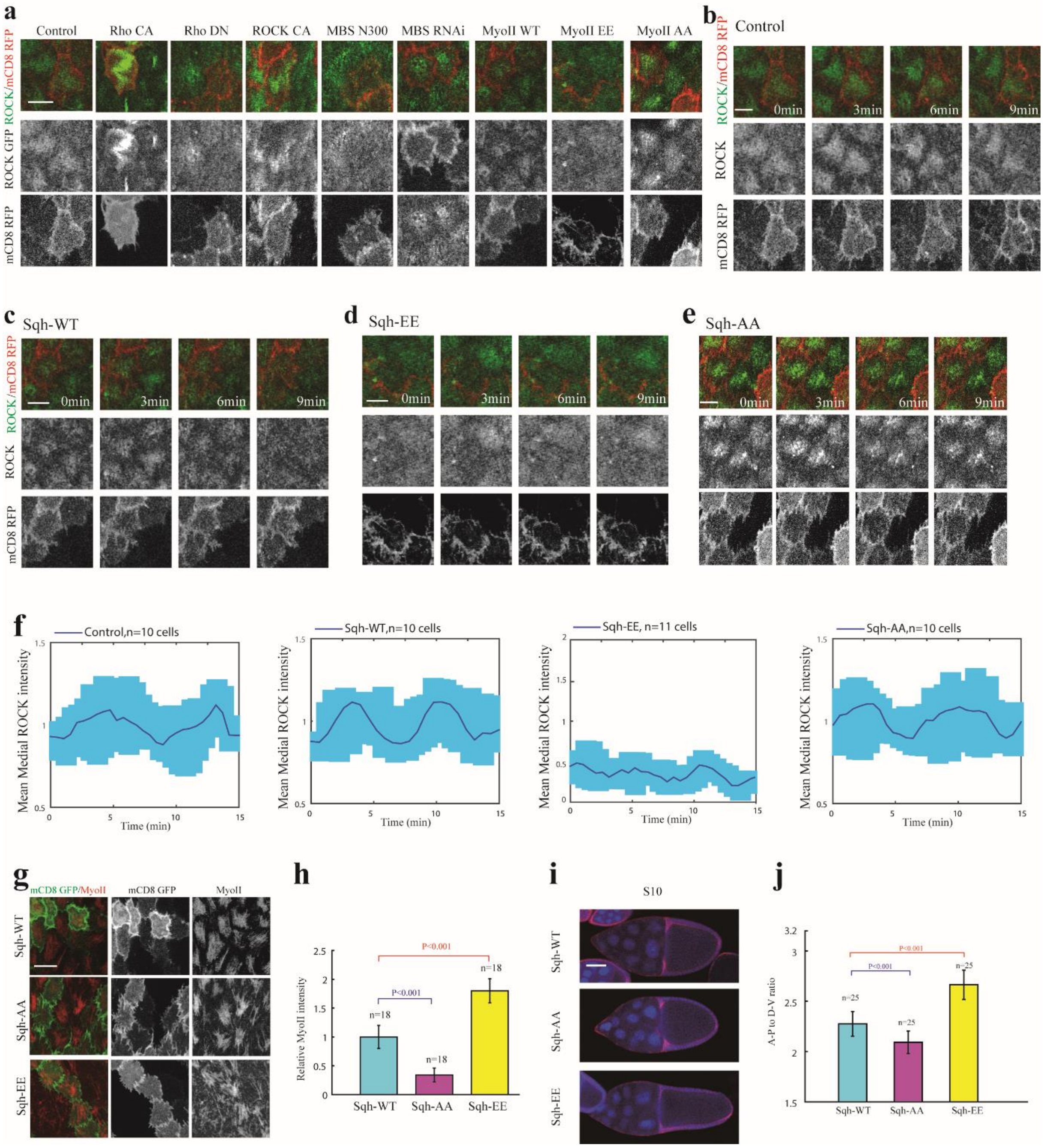

Supplementary Figure 3. The positive and negative regulators in control of ROCK-GFP dynamic intensity

a. Basal view of follicle cell clones expressing the indicated transgenes, marked by coexpression of mCD8RFP. ROCK signals are monitored by ROCK-GFP. **b-e.** Time-lapse series of ROCK-GFP signals in follicle cell clones expressing the indicated transgenes, labelled with mCD8-RFP. **f.** Quantifications of the dynamic change of medial basal mean ROCK intensity in the indicated transgene-expressing cells. Each dark blue curve is the dynamic change from the average of ROCK intensities in different cells with a range marked by blue color. **g.** Basal views of follicle cell clones expressing the indicated transgenes, marked by coexpression of mCD8GFP. Signals of MyoII have been assessed by MyoII-mCherry. **h.** Quantification of relative MyoII intensity in the indicated transgene-expressing

GFP-positive cells compared with the GFP-negative wild type cells in the same sample. **i.** Morphology of stage-10 egg chambers expressing the indicated transgenes, staining by Armadillo and DAPI, 4',6-diamidino-2-phenylindole. **j.** Quantification of the A-P to D-V length ratio in the stage-10 egg chambers expressing the indicated transgenes. n is the number of samples analyzed. Scale bars are 10 μm in (**a-e, g**) and 50 μm in (**i**). Error bars indicate ±s.d. $p<0.001$ means significant difference by student's *t*-test.

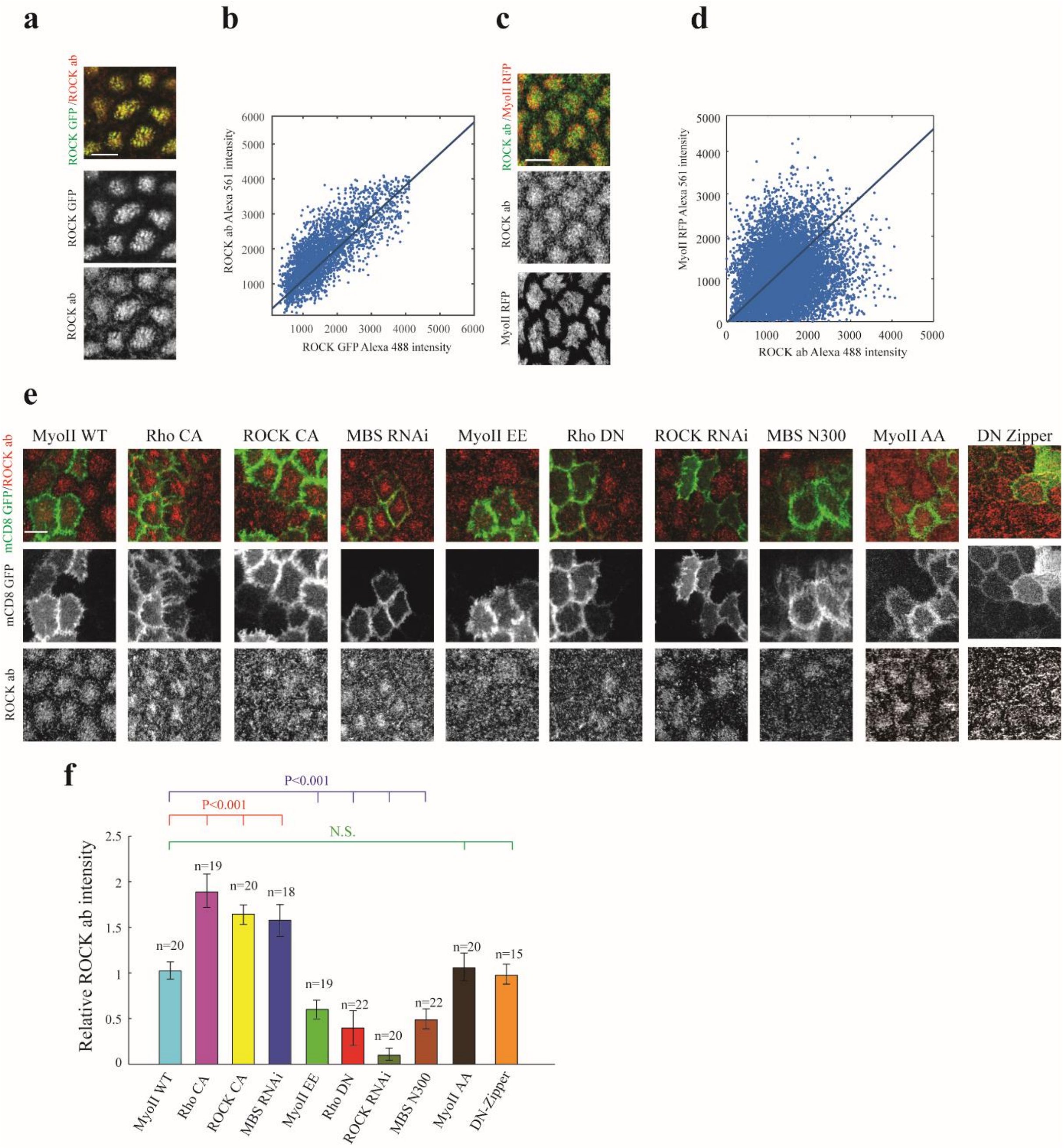

Supplementary Figure 4. Confirmation of the ROCK antibody efficiency and the positive and negative regulators in control of endogenous ROCK intensity

a, c. Confocal micrographs of follicle cells expressing ROCK-GFP (**a**) or MyoII-mCherry (**c**), stained with ROCK antibody. **b, d.** Similarity comparisons between ROCK-GFP (**b**) or MyoII-mCherry (**d**) and ROCK antibody staining. **e.** Basal view of follicle cell clones expressing the indicated transgenes, marked by coexpression of mCD8GFP. Endogenous ROCK signals are monitored by the ROCK antibody staining. All scale bars are 10 µm. **f.** Quantification of relative ROCK intensity in the indicated transgene-expressing GFP-positive cells compared with wild type cells in the same tissue sample. n is the number of samples analyzed. All error bars indicate ±s.d. N.S means no significant difference, while p<0.001 means significant difference by student's *t*-test.

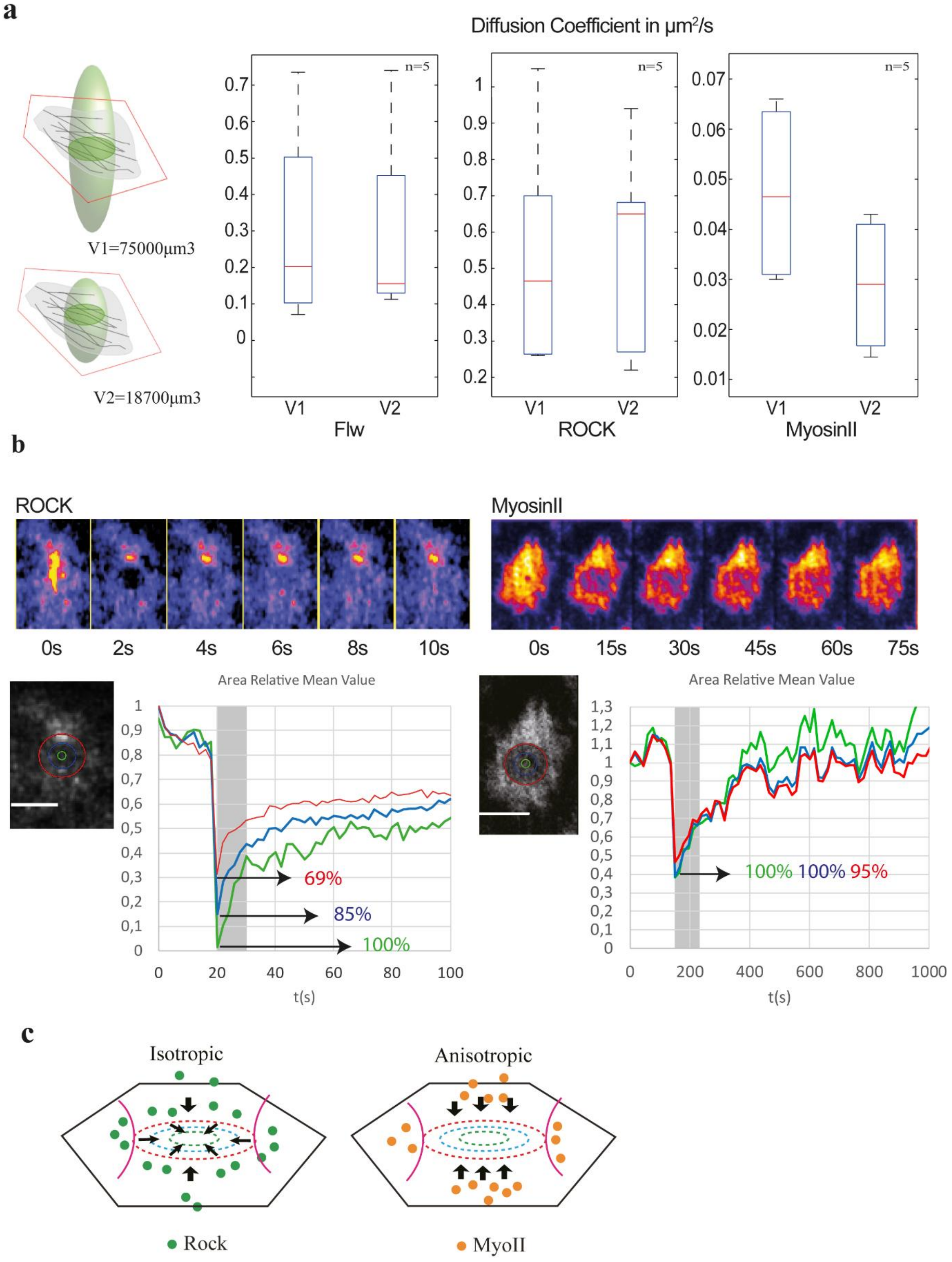

Supplementary Figure 5. ROCK and Flw signals have very different diffusion characteristics from those of Myo-II signals

a. FRAP analysis with variable 3D confocal volumes, for ROCK-GFP, Flw-YFP and MyoII-

GFP signals. Left panels are schematics of two different 3D volumes of follicle cells near basal domain. Right panels show the diffusion coefficients of the indicated signals, analyzed by FRAP at these two different 3D volumes, and in particular that Myo-II recovery is more strongly affected by these two different 3D volumes, than ROCK and Flw recovery. **b.** Characteristic time series and signal recoveries following FRAP experiments of each component of the biochemical oscillation. Upper panels show the time-lapse series of ROCK-GFP (left) and MyoII-GFP (right) signals before and after photobleaching. Lower panels show the recovery curve of ROCK-GFP (left) and MyoII-GFP (right) signals after photobleaching, at 3 circle regions labelled by green, blue, and red colour circle (these 3 regions represent the inside, middle and outside sections of the photobleached regions). Both scale bars are 5μm. The shade region in the recovery curve is the time periods in which the snapshots of ROCK-GFP and MyoII-GFP signals have been shown in the upper panel. For ROCK signals, intensity reduction after photobleaching detected at the inside region is maximal and the intensity recovery speed is slowest; while the intensity reduction after photobleaching detected at the middle and outside regions is smaller (85% and 69% of intensity reduction at the middle and outside regions, respectively, relative to 100% of intensity reduction detected at the inside region), and the recovery speed is faster. Flw-YFP signals have the similar intensity recovery characteristics to those of ROCK signals (data not shown). But for Myo-II signals, there is no significant difference of the intensity reduction after photobleaching detected at these 3 regions, and the recovery speed is similar too. These 2D diffusion analyses indicate that Myo-II diffusion occurs mostly in a 3D manner, while ROCK diffusion has some 2D characteristics. **c.** Schematic cartoon to summarize two different characteristics of ROCK and Myo-II diffusion. Anisotropic diffusion of Myo-II signals is mainly from 3D turnover, while some isotropic diffusion of ROCK signals is from 2D turnover (although we cannot exclude the effect of 3D turnover).

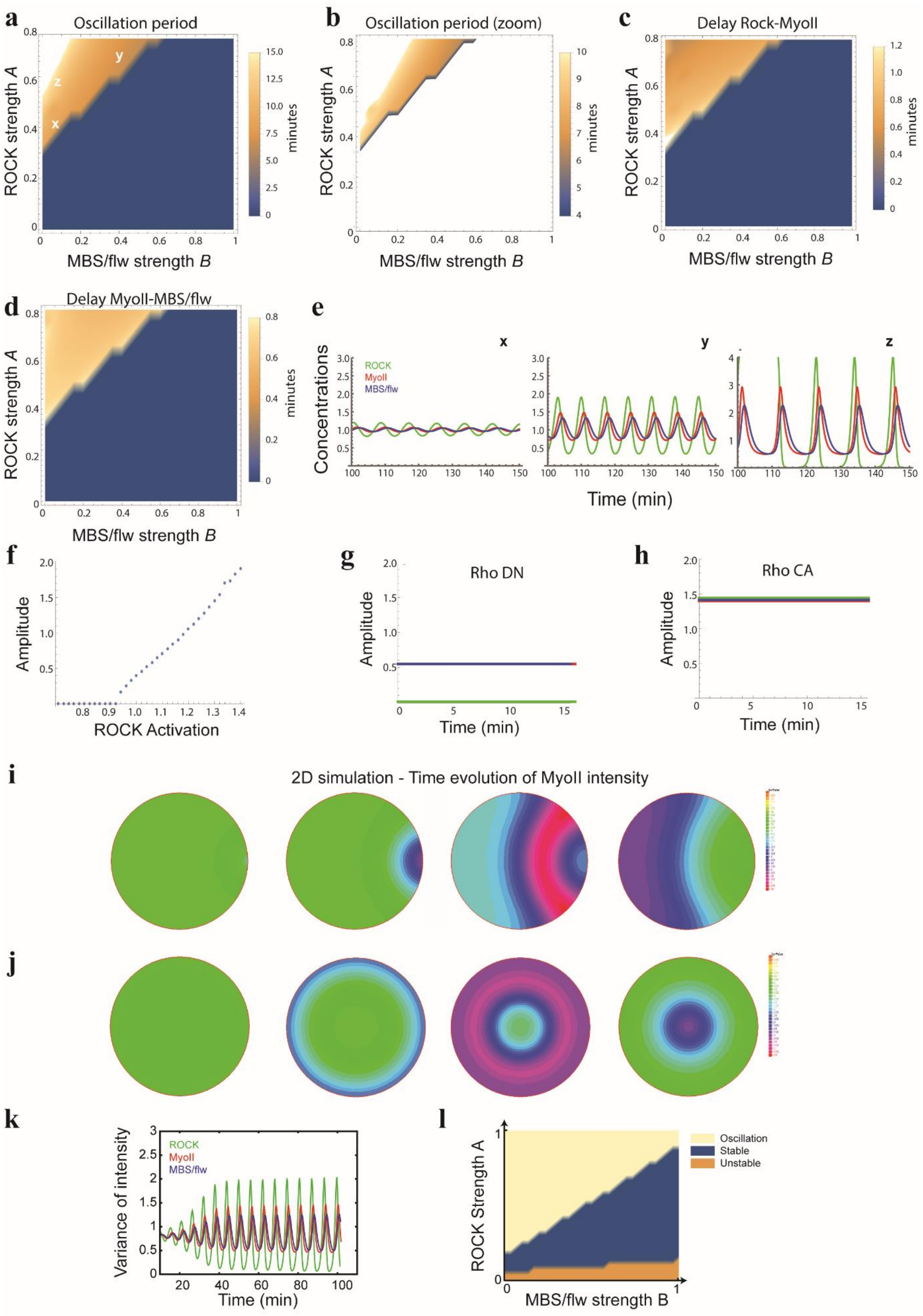
a
Oscillation period
ROCK strength A
MBS/flw strength B
minutes
b
Oscillation period (zoom)
c
Delay Rock-MyoII
d
Delay MyoII-MBS/flw
e
Concentrations
ROCK
MyoII
MBS/flw
x
y
z
Time (min)
f
Amplitude
ROCK Activation
g
Rho DN
Time (min)
h
Rho CA
i
2D simulation - Time evolution of MyoII intensity
j
k
Variance of intensity
Time (min)
l
ROCK Strength A
MBS/flw strength B
Oscillation
Stable
Unstable

Suppementary Figure 6. ***In silico*** **prediction of the activator-inhibitor intensity effects on basal Myo-II oscillation cycle time period and signal delay time**

a-d. Sensitivity analysis for Figure 5, showing how the oscillation period **(a, b),** Rock-MyoII delay **(c)** and MyoII-MBS/Flw delay **(d)** change depending on the relative strength of the interaction of ROCK and MBS/Flw on Myo-II (A and B in the model). We show that oscillations (defined either by a numerical integration of the equations, or by a pair of conjugate eigenvalues with positive real part) occur above a threshold of ROCK strength compared to MBS/Flw strength, and that we predict values for period and delays which are robustly close to experiments. **e.** Examples of numerical solutions in the oscillatory region of the phase diagram, denoted in panel **a.** (from left to right) as "x" *(A=0.425, B=0.1)*, "y" *(A=0.7, B=0.4)* and "z" *(A=0.6, B=0.1)*. **f.** Predicted amplitude of the oscillation as a function of the auto-activation strength of ROCK (as computed in Figure 5d-g), showing a transition towards oscillations above a critical value. **g.** Simulated experiment of dominant negative Rho, which we assume divides by half the auto-activation property of ROCK, causing a loss of oscillations (see panel **f**). **h.** Adding a constant influx of ROCK activation (independent of ROCK activation status, to mirror constitutively active Rho) is predicted to destroy oscillations above a given threshold from a theoretical perspective. **i, j.** Snapshots from a numerical simulation on the same system of equation, but in 2D on a disk with no-flux boundary conditions, with color-coded Myo-II intensity (green corresponding to low concentration and pink to high concentration). Panel **i.** shows a numerical integration with preferential activation of ROCK at a restricted region of the rim (around $\theta=0$), whereas panel **j.** shows a numerical integration with preferential activation of ROCK all around the disk rim. All parameters are the same as in the 1D simulation of Figure 5. **k.** Variance of concentrations (ROCK, Myo-II and MBS/Flw in green, red, and blue resp.) as a function of time from the integration shown in panel **j.** indicates that oscillations occur within a similar sequence and period as in the non-dimensional case. **l.** Phase diagram for the system as a function of the relative strength of the interaction of ROCK and MBS/Flw on Myo-II (A and B in the model), when including an additional negative feedback of ROCK of MBS/Flw ($\alpha=0.1$, see Supplementary Note, Section 1.3 for details).

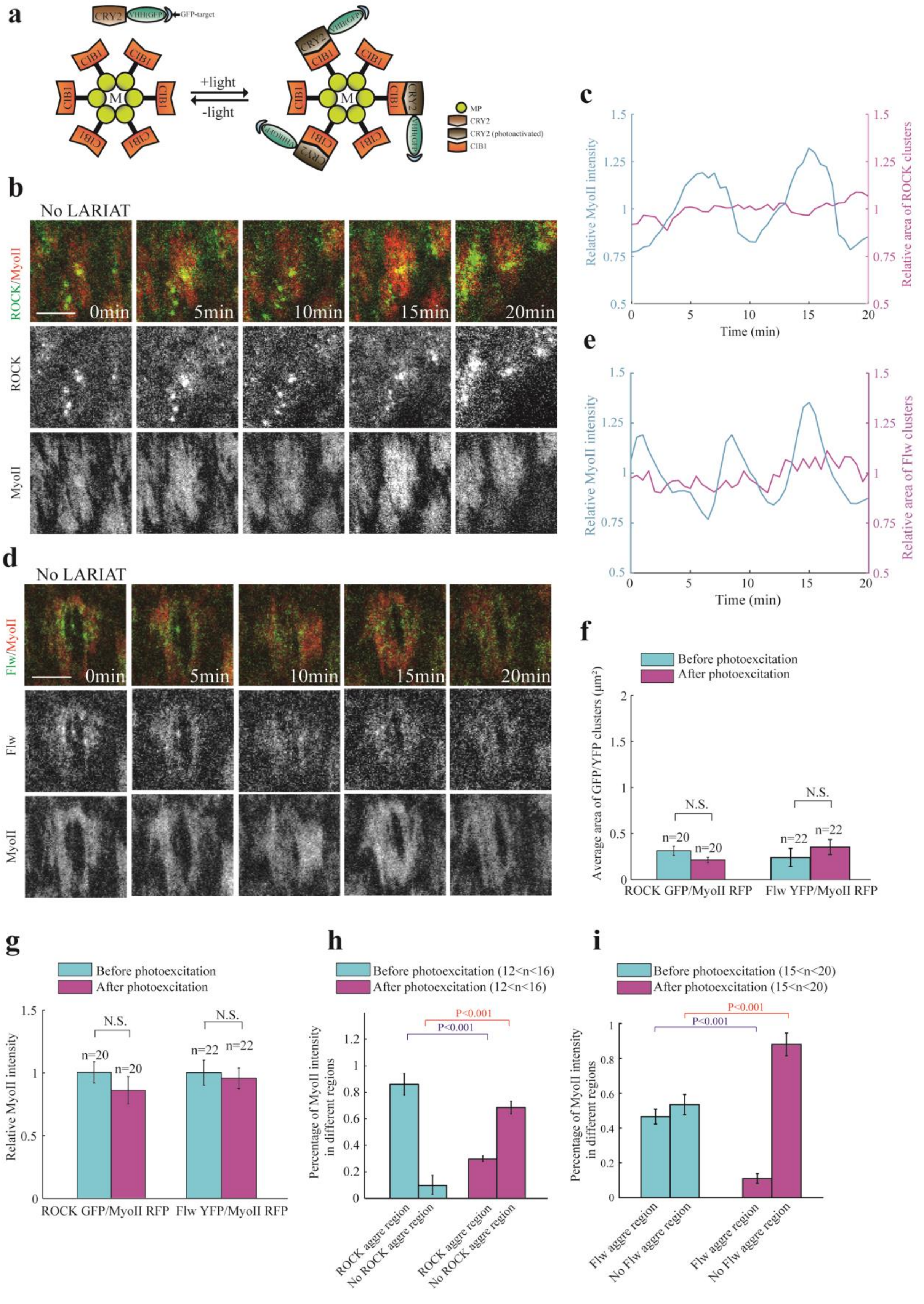

Supplementary Figure 7. Basal Myo-II oscillations are not affected by light illumination

in the no LARIAT-expressing follicle cells

a. Schematic of the blue light-mediated GFP cluster formation. **b, d.** Time-lapse series of the representative no LARIAT-expressing follicle cells, labelled with ROCK-GFP and MyoII-mCherry (**b**) and Flw-YFP and MyoII-mCherry (**d**), under photoexcitation. Both scale bars are 5 μm. **c, e.** Quantifications of the dynamic change of relative Myo-II intensity and relative area of ROCK clusters (**c**) and Flw clusters (**e**) in no LARIAT-expressing follicle cells, under photoexcitation. **f, g.** Quantifications of average GFP/YFP clustering area (**f**) and relative Myo-II intensity (**g**) before and after photoexcitation in no LARIAT-expressing follicle cells. **h, i.** Quantifications of relative Myo-II intensity in different regions (total region, ROCK aggregation region, and no-ROCK aggregation region; total region, Flw aggregation region, and non-Flw aggregation region) of the LARIAT-expressing follicle cells, labelled with ROCK-GFP (**h**) and Flw-YFP (**i**), before and after photoexcitation, whose corresponding representative time-lapse images have been shown in Figure. 7e, f. n is the number of samples analyzed. Error bars indicate ±s.d. N.S means no significant difference, $p<0.001$ means significant difference by student's *t*-test.

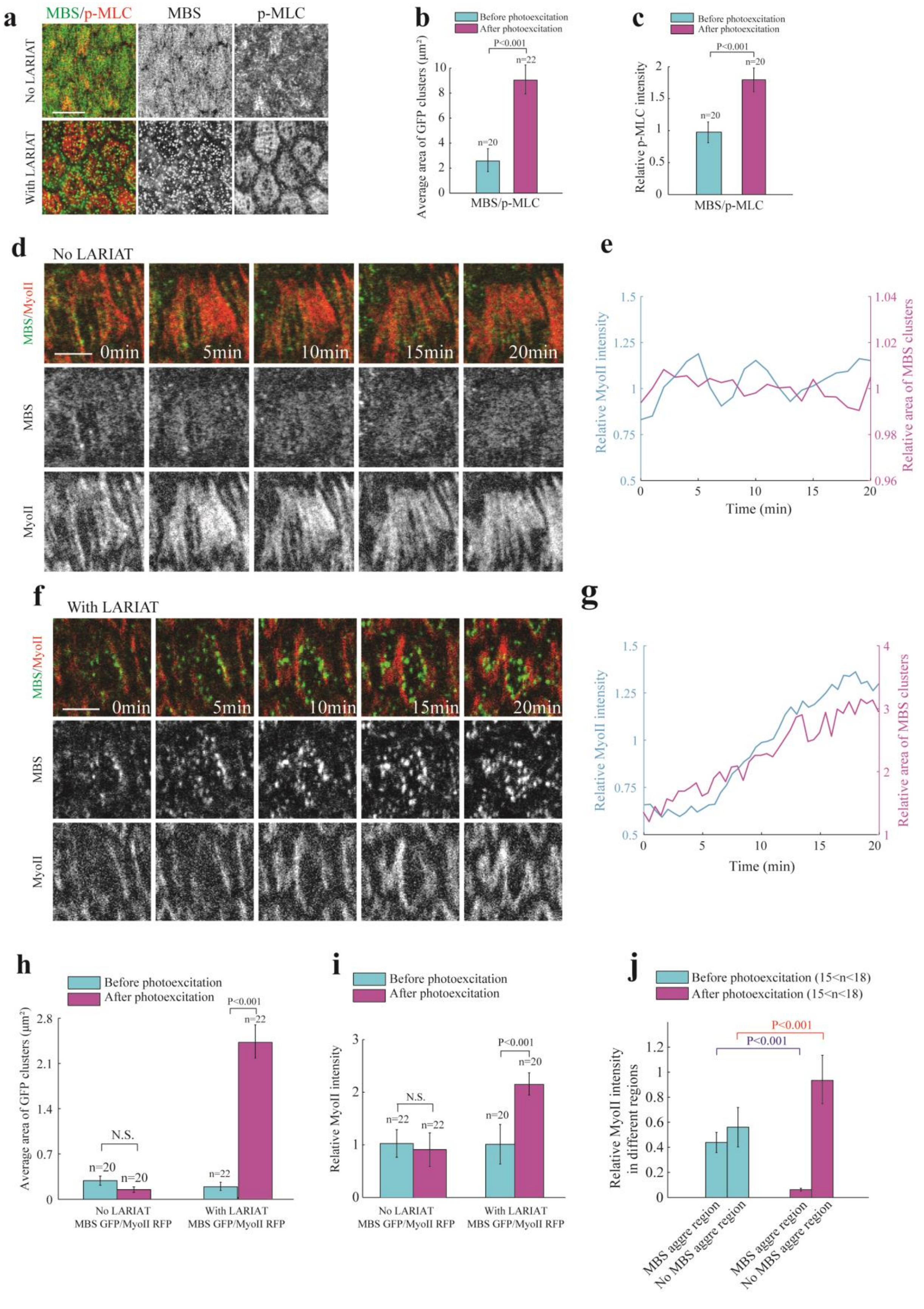

Supplementary Figure 8. Basal Myo-II oscillations are enhanced by MBS-GFP clustering

a. Basal view of MBS-GFP, together with p-MRLC signal marked by p-MRLC antibody staining in the LARIAT-expressing follicle cells, under the dark condition or after 1 hour photoexcitation by visible light. Both Scale bar is 10 μm. **b, c.** Quantifications of average GFP clustering area (**b**) and relative p-MRLC intensity (**c**) before and after photoexcitation in the LARIAT-expressing follicle cells. **d, f.** Time-lapse series of the representative no LARIAT-expressing (**d**) and LARIAT-expressing (**f**) follicle cells, labelled with MBS-GFP and MyoII-mCherry, under photoexcitation. Both scale bars are 5 μm. **e, g.** Quantifications of the dynamic change of relative Myo-II intensity and relative area of ROCK clusters in the no LARIAT-expressing (**e**) and LARIAT-expressing (**g**) follicle cells, under photoexcitation. **h, i.** Quantifications of average MBS clustering area (**h**) and relative Myo-II intensity (**i**) before and after photoexcitation in the no LARIAT-expressing and LARIAT-expressing follicle cells. **j.** Quantifications of relative Myo-II intensity in different regions (total region, MBS aggregation region, and no-MBS aggregation region) of the LARIAT-expressing follicle cells, labelled with MBS-GFP, before and after photoexcitation. n is the number of samples analyzed. Error bars indicate ±s.d. N.S means no significant difference, $P<0.001$ means significant difference by student's *t*-test.

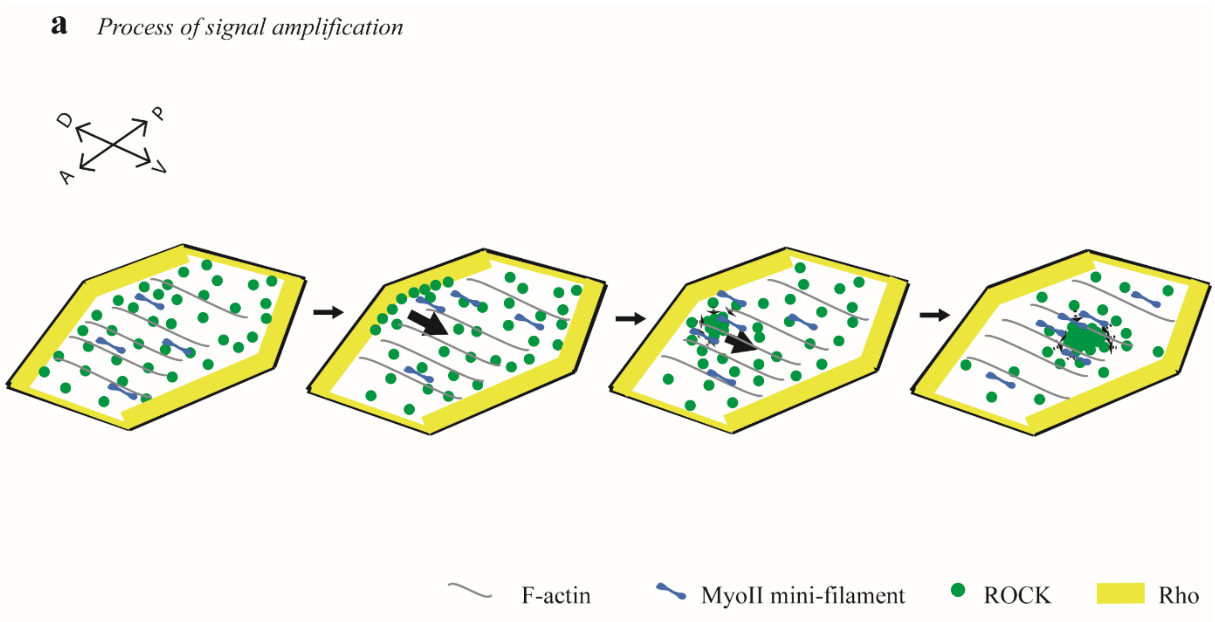

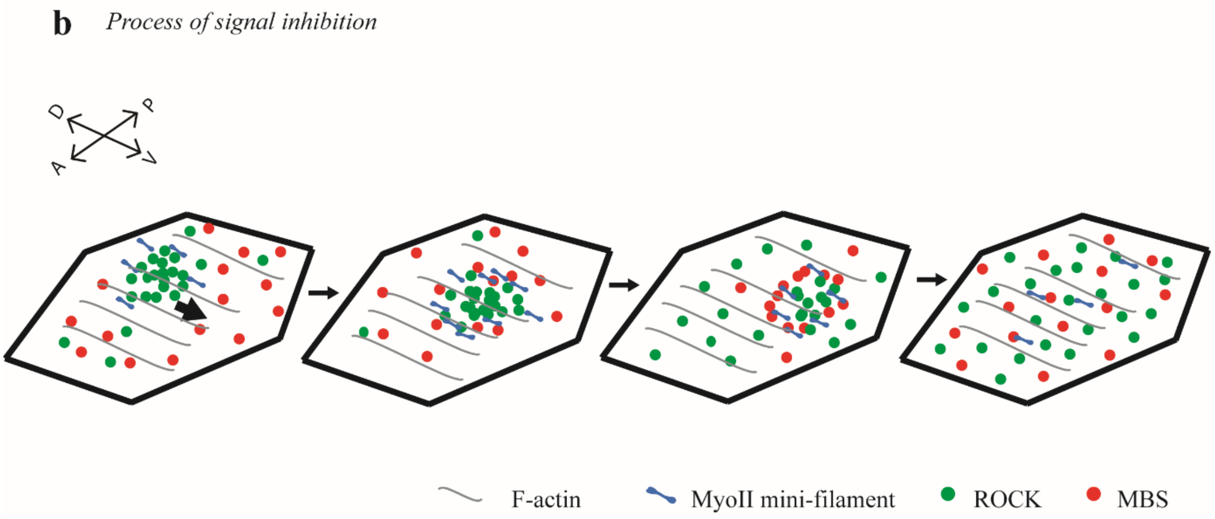

Supplementary Figure 9. Model of basal Myo-II oscillation

a. Schematic cartoon to summarize the process of signal amplification. Firstly, ROCK interacts with basal junctional Rho1 to be activated; then activated ROCK flows from junctional membrane to medial basal cortex; during this flow movement, ROCK signals get self-amplified by the kinase activity-dependent ROCK accumulation, and ROCK also activates Myo-II, thus leading to Myo-II accumulation on actin filaments. **b.** Schematic cartoon to summarize the process of signal inhibition. During the flow movement of ROCK, the locally accumulated Myo-II distributed at the medial basal cortex redistributes MBS (MLCP) from diffusion status to the local enrichment around Myo-II; this locally enriched MLCP activity is able to shut off both ROCK and Myo-II accumulation signals, thus finally leading to the disassembly of all signals.

Supplementary Note 1: Methods of FRAP Analysis for diffusion fittingWe used the methods

propose from embl *FrapCalc* software for quantitative analysis of frap data.
https://github.com/framasoft/framacalc

1- Region of Interest used for analysis.

The process of analysis used four different regions for each experiment (see Supplementary Figure 10 and 11).

1- FRAP ROI (ROI=Region Of Interest). Region where you actually bleached the fluorophore by strong laser irradiation. Shape and size are circular, and correspond to Shape of the FRAP ROI, circular, rectangular, affects the model equation to be fitted with. Size of the ROI matters with the diffusion-limited type of FRAP recovery (see below).

2- Base ROI Also called 'background'. Set ROI outside the cell, where there should be no fluorescence, to know the offset intensity.

3- *Whole Cell ROI* or 'All cell'. The average fluorescence intensity of the whole cell you are observing. For myosin oscillation, the mean intensity measurements is corrected by another RED channel without photobleaching to avoid chemical-oscillation phenomenon [1].

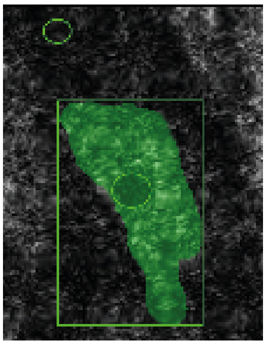

Supplementary Figure 10: exemple of ROI used for frap analysis during myosin oscillation

2- Normalization of the FRAP curve

We used the ratio of medial MyoII in red and green channels was used to do the above normalization. In that case, we can hope to analyse pure diffusion phenomenon. Next, we used the known methods called the double normalization [2] .We take the measurement from whole cell ROI for correcting the acquisition bleaching effects. Average pre-bleach whole cell intensity divided by the whole cell intensity at each time points in the post-bleach period will be multiplied to the FRAP curve at that time point. Before this operation, both Whole Cell ROI and FRAP ROI data are subtracted by Base ROI intensity.

$$I_{frap-norm}(t) = \frac{I_{frap-norm}(t) - I_{frap-bleach}}{I_{frap-pre} - I_{frap-bleach}}$$

Supplementary Figure 11: Exemple of normalization and fit of diffusion from ROCK

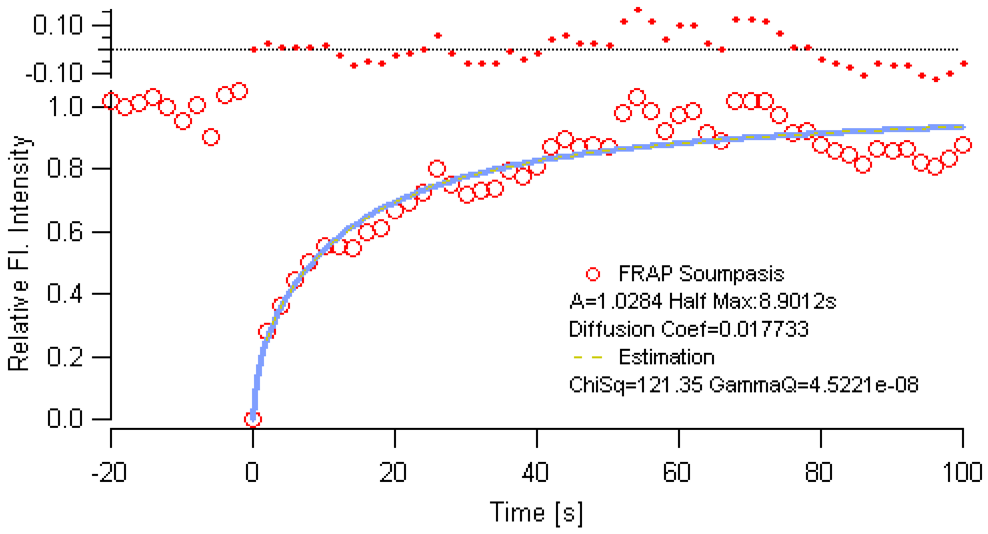

Fraction mobile and Half Max is estimate after Normalization with a PHAIR Double Exponential Fitting by the following formula.

$$I_{frap-post}(t) = y_0 - A_1 e^{\tau_1 t} - A_1 e^{\tau_2 t}$$

Mobile fraction can be estimated like:

$$Mob = \frac{-(A_1 + A_2)}{1 - (y_0 + A_1 + A_2)}$$

2- Fitting of diffusion mecanism

We fit double-normalized FRAP curve with the well-known Ellenberg Diffusion Fitting empirical formula

$$I(t) = I_{final}\left(1 - \left(\frac{w^2}{w^2 + 4\pi D t}\right)^{1/2}\right)$$

as proposed by Ellenberg [3].

w is the width of the strip-bleaching and need a user input. The fitting will look for a likely two parameters I_{final} and D, the diffusion coefficient. I_{final} will be considered as the mobile fraction. Half max can be calculated in μ²m/s as

$$\tau_{1/2} = \frac{0.75 w^2}{\pi D}$$

Supplementary Note 2

In this Supplementary Note, we provide additional details on our theoretical model of non-muscle Myosin II (MyoII) oscillations in the basal cortex of *Drosophila* oocytes. Several experimental data (Fig. 3) underline that ”mechanical” interactions of the actomyosin network are dispensable for the oscillations, contrary to several proposed models in various systems. Instead, we thus explore in this Theory Note the possibility of biochemical oscillations, and thus only write chemical conservation equations for each of the key component of the MyoII regulatory pathway.

1 Zero-dimensional model

In this first section, we begin by neglecting any spatial dependency of concentration, and seek to gain physical insight into the oscillations by starting through a zero-dimension model. The general ordinary differential equations (without delays) one can write[4] of Rock, MyoII and MBS/flw concentrations (resp. R, m and M), taking into account all possible couplings between each species are:

$$\begin{cases} \frac{dR}{dt} = f(R,m,M) \\ \frac{dm}{dt} = g(R,m,M) \\ \frac{dM}{dt} = h(R,m,M) \end{cases} \tag{1}$$

f, g and h are functions which do not depend on the past history of the system (Markovian process), but only on the concentration of each species. In the following subsections, we show that our experimental data from the main text, as well as the past literature, put considerable constraints on each of these functions. We review both the positive and negative interactions between species, as well as the possibility of self-activation and self-inhibition of each given specie.

1.1 Reported interactions

Positive feedback mutual interactions
From the literature, it is clear that Rock activity phosphorylates MyoII light chain, leading to MyoII recruitment and assembly into MyoII filaments[5–8]. This puts the constraint $\frac{\partial g}{\partial R} > 0$ on the system of equations (1). We validated this in our system by optogenetic entrapment of ROCK, which causes the concomitant decrease in MyoII intensity (Fig. 7e,g). Moreover, it has also been shown that MyoII is a substrate for MBS, which possesses a MyoII binding site[9]. This puts the constraint $\frac{\partial h}{\partial m} > 0$ on our system of equations.

Negative feedback mutual interactions
From the literature, it is also clear that MBS/Flw activity de-phosphorylates MyoII, leading to its dis-assembly and concentration decrease ($\frac{\partial g}{\partial M} < 0$), from biochemical studies[10], but also in cultured cells[11] as well as *in vivo* in *Drosophila*[12–13]. We validated this in again in our system by optogenetic entrapment of Flw or MBS, which causes a concomitant increase in MyoII concentration (resp. Fig 7f,h-k and Supplementary Fig.8).

Self-activation and protein turnover
Finally, ROCK has been shown in biochemical studies to be able to perform trans- and auto-phosphorylation, raising the possibility of a self-activatory loop[5,14]. Although rarely discussed in the context of MyoII oscillations, this property of ROCK is seductive for our modelling purposes, as self-activation is known to generically allow for oscillatory behaviours (the most

famous class being the Turing activator-repressor). As discussed in the main text, we therefore sought to test the possibility of ROCK self-activation in different ways. In particular, we expressed either expressed a constitutively active form of ROCK, or downregulated its expression via ROCK RNAi, and observed that this increased and decreased, respectively, the concentration of endogenous ROCK, which provides a key line of evidence for a positive self-activation loop.

On the other hand, based on previous literature, MyoII cannot self-assemble without a positive signal phosphorylating its light chain[7], this translates into $\frac{\partial g}{\partial m} < 0$, i.e. without ROCK-mediated phosphorylation, myosin concentration would drop drastically. However, as MyoII has been shown to be phosphorylated by ROCK-independent pathways, such as MLCK[15] or MRCK[6], we include a small, constant, contribution $J > 0$, acting as a source term and contributing positively to MyoII activation and recruitment.

1.2 Proposed interactions

Anticipating slightly on the following sections, one can see that the above set of interactions would not yield to ROCK oscillations, as we have not provided a negative feedback on ROCK yet. Therefore, ROCK concentration would simply reach its steady-state monotonically and remain constant, contrary to our observations. This hints to the idea that a negative feedback to ROCK must exist. As we show in the main text that Rho signalling, which activates ROCK[5] does not show a pulsatile behavior, similar to results observed in the apical medial pool in the *Drosophila* embryo[1], we explore experimentally alternative feedbacks arising from MyoII. In particular, we show (Fig. 5a) in the main text that MBS constitutive activation or down-regulation respectively decreases or increases drastically ROCK concentration, consistent with a negative feedback of MBS on ROCK. This feedback could either by direct through dephosphorylation by MBS of ROCK, or via a more complex molecular cascade. However, for the sake of simplicity, we model this effect via a direct negative feedback of MBS on the reaction/activation rate of ROCK ($\frac{\partial f}{\partial M} < 0$). Interestingly, it has been reported that MBS contains binding sites for RhoA[8].

1.3 Model and non-dimensionalization

Given the constraints described above, we can therefore re-write our set of equations in a generic manner as:

$$\begin{cases} \frac{dR}{dt} = \frac{R}{\tau_R}(1 - \alpha_1 M) \\ \frac{dm}{dt} = -\frac{m}{\tau_m}(1 + \alpha_2 M - \alpha_3 R) + \frac{J}{\tau_m} \\ \frac{dM}{dt} = \frac{\alpha_4 m - M}{\tau_M} \end{cases} \tag{2}$$

where we have assumed first-order kinetics for the turnover rates. τ_R, τ_m and τ_M are characteristic timescale associated with the dynamics of respectively ROCK, MyoII and MBS. α_i, $i \in [1,4]$ are strictly positive coefficients associated with the interactions between ROCK, MyoII and MBS described above. Here, for the sake of simplicity, we have modelled Rock auto-activation as a linear term, following the convention from the classical FitzHugh-Nagumo model (as a prototype of activator/inhibitor kinetics in the spirit of Turing). One should note that we could have in principle included additional non-linear terms, for instance describing the saturating kinetics of ROCK auto-activation, using Hill functions[4]. Nevertheless, as we will see in the following sections, the interactions we have specified above are enough for the system for reach consistently a stable equilibrium with positive, non-zero concentrations. Therefore, for the sake of parsimony, we do not consider such higher-order terms, which would not change the key theoretical behaviors that we describe here. Similarly, we could also have modelled

the direct inhibition of MBS activity by ROCK[8,16], which has been shown conclusively for ROCK1 in cultured cells (although ROCK2 does not show such a direct interaction)[17]. However, whether *rok* directly interacts with MBS in *Drosophila* is still unclear[18], and would not change again qualitatively our modelling result. Indeed, we carried out the stability analysis of including a feedback of strength α of ROCK on MBS: $\frac{dM}{dt} = \frac{-\alpha R + \alpha_4 m - M}{\tau_M}$. Importantly, when studying the eigenvalues of the new system of equations, we found that the transition from an homogeneous to an oscillatory solution (i.e. a pair of conjugate eigenvalues with a positive real part) was qualitatively unaffected (occurring above a threshold for A which increased with B). However, as seen on Supplementary Fig. 6I (for $\alpha = 0.1$), such a feedbackproduces a second zone of instability, characterised by a single real eigenvalue becomingpositive (loss of stability of the homogeneous solution without oscillations), below a criticalvalue of A. However, in thiscase, we found that additional non-linearities would benecessary to prevent negative values of concentrations. As this does not produce oscillatorysolutions, we thus did not explore this feedback further.

In the system of equations above, the feedbacks on MBS and ROCK (first two equations) intervene by changing the reactions rate in the Rock and MyoII equations, as they change the phosphorylation status of both proteins, thus modifying their aggregation/dis-assembly properties. On the other hand, the feedback on MBS (third equation) intervenes as a source term, consistent with the aforementioned role of MyoII as a substrate for MBS. We first start by determining the steady-state of 2.

$$\begin{cases} M_0 = 1/\alpha_1 \\ R_0 = \frac{\alpha_1 + \alpha_2 - \alpha_1^2 \alpha_4 J}{\alpha_1 \alpha_3} \\ m_0 = 1/(\alpha_1 \alpha_4) \end{cases} \tag{3}$$

One should note that Eq. (3) imposes an upper-limit on the value of the source term J, as the concentration of Rock cannot become negative, and all other coefficients are defined positive. As it is not possible to compare absolute concentrations of our different species experimentally, we simplify our set of equation by using dimensionless variables $R' = R/R_0$, $m' = m/m_0$, $M' = M/M_0$, and drop the prime superscripts for the sake of simplicity in the following.

We can then re-write Eq. (2) as:

$$\begin{cases} \frac{dR}{dt} = \frac{R}{\tau_R}(1 - M) \\ \frac{dm}{dt} = -\frac{m}{\tau_m}(1 + BM - AR) + \frac{1+B-A}{\tau_m} \\ \frac{dM}{dt} = \frac{m - M}{\tau_M} \end{cases} \tag{4}$$

with $B = \frac{\alpha_2}{\alpha_1}$ and $A = \frac{\alpha_1 + \alpha_2 - \alpha_1^2 \alpha_4 \tau_m J}{\alpha_1}$. Therefore, the non-dimensional equations governing the kinetics of Rock, MyoII and MBS involve 4 rescaled parameters: 2 rescaled timescales comparing the dynamics of each species, and 2 rescaled interaction coefficients A and B.

As three-species chemical systems are much more cumbersome to treat analytically than two-species chemical systems, one would be tempted to try to find a limit in which the system (4) reduces to two effective species. Nevertheless, as we show in the main text, the period of the oscillations and the delays between each species is on the same order of magnitude, which prevents us from making such simplified assumptions.

Nevertheless, it is still useful to consider, in the following sections, different limits to our system, in order to gain intuition into the physics of the oscillations.

1.4 Two-species limiting cases

1.4.1 Infinitely fast relaxation of MBS

We first start by assuming $\tau_M \to 0$, i.e. MBS relaxes extremely rapidly, so that its concentration is simply proportional and slave to the concentration of MyoII.

$$\begin{cases} \frac{dR}{dt} = \frac{R}{\tau_R}(1-m) \\ \frac{dm}{dt} = -\frac{m}{\tau_m}(1+Bm-AR) + \frac{1+B-A}{\tau_m} \end{cases} \tag{5}$$

We thus see that the system belong to the equivalence class of activator-inhibitor systems, one of the four canonical cases of two-species oscillators studied by Turing[19]: Rock activates itself and upregulates MyoII, while MyoII inhibits itself as well as Rock (via MBS). System (7) resembles in particular the FitzHugh-Nagumo model for action potential propagation in neurons[20], with zero external stimulus, although the non-linearities are slightly different. It can also be seen as a generalization of the two-species competitive Lotka-Volterra[21] model with a source term $J_0 = 1+B-A$.

The concentrations in the homogeneous solution are then $m_0 = 1$ and $R_0 = 1$. A linear stability analysis on the variables $\delta R = R/R_0 - 1$ and $\delta m = m/m_0 - 1$ yields the Jacobian matrix

$$M = \begin{pmatrix} 0 & -\frac{1}{\tau_R} \\ \frac{A}{\tau_m} & -\frac{1+2B-A}{\tau_m} \end{pmatrix} \tag{6}$$

In the general case, the eigenvalues are given by

$$2\lambda_{1,2} = -\frac{1+2B-A \pm \sqrt{(1+2B-A)^2 - 4A\tau_m/\tau_R}}{\tau_m}$$

The system goes from a homogeneous to a temporally periodic solution when these eigenvalues are complex conjugate whose real part is positive. Therefore, we predict oscillations if $A > 1+2B$, i.e. if the positive regulation overcome the negative regulation. One should note that in this two-species setting, one needs to relax the assumption of $J_0 > 0$ (introduced for the three-species system) in order to get oscillations. The period which can be deduced from the linear stability analysis is $T = \sqrt{\frac{\tau_m \tau_R}{A}}$, and is therefore proportional to the geometric mean of the two characteristic time scales associated with the two species. Nevertheless, the period of the real limit cycle can be different from the one deduced from the linear stability analysis given that the fixed point is not hyperbolic, and must therefore be deduced in the general case from numerical integrations. In particular, numerical integration of this system reveals that one would require additional non-linearities to prevent the concentrations from becoming negative.

1.4.2 Infinitely fast relaxation of MyoII

We then continue by assuming $\tau_m \to 0$, i.e. MyoII relaxes extremely rapidly, so that its concentration is simply proportional and slave to the concentration of MBS and Rock: $m = \frac{1+B-A}{1+BM-AR}$:

$$\begin{cases} \tau_R \frac{dR}{dt} = R(1-M) \\ \tau_M \frac{dM}{dt} = \frac{1+B-A}{1+BM-AR} - M \end{cases} \tag{7}$$

The concentrations for the homogeneous solution are then $M_0 = 1$ and $R_0 = 1$. A linear stability analysis on the variables $\delta R = R/R_0 - 1$ and $\delta M = M/M_0 - 1$ yields the Jacobian matrix

$$M = \begin{pmatrix} 0 & -1/\tau_R \\ \frac{1}{\tau_M}\frac{1}{1+B-A} & -\frac{1}{\tau_M}(1+\frac{1}{1+B-A}) \end{pmatrix} \quad (8)$$

which cannot not have complex eigenvalues with positive real parts. Indeed, assuming complex conjugate eigenvalues of the form $\lambda_{1,2} = \lambda \pm iw$ (with $w \in \mathbb{R}$), and taking $\lambda = 0$ at the critical point leads to $w^2 = -\frac{1}{\tau_R \tau_M}$, a contradiction. This therefore indicates that the finite relaxation time of MyoII is crucial for the presence of oscillations in this model.

1.5 Three species limiting-cases

Similar to the subsection above, a linear stability analysis on the variables δR, δm and δM yields the Jacobian matrix in the generic case:

$$M = \begin{pmatrix} 0 & 0 & -1/\tau_R \\ \frac{A}{\tau_m} & -\frac{1+B-A}{\tau_m} & -\frac{B}{\tau_m} \\ 0 & 1/\tau_M & -1/\tau_M \end{pmatrix} \quad (9)$$

As the analytical expression of the eigenvalues is rather complicated, we make here the simplifying assumption that all rates are identical: $\tau_R = \tau_m = \tau_M = T$, again to gain analytical insight into the problem. One simplifying feature in particular is that the threshold of the instability is then independent on T.

We are looking for the appearance of a periodic solution, i.e. that matrix M has one real eigenvalue, and two complex conjugate eigenvalues:

$$M_d = \begin{pmatrix} \lambda_1 & 0 & 0 \\ 0 & \lambda + iw & 0 \\ 0 & 0 & \lambda - iw \end{pmatrix} \quad (10)$$

For $\lambda < 0$, the stationary solution is stable, whereas for $\lambda > 0$, a periodic solution appears. Identifications of the characteristic polynomials of M and M_d, together with the condition $\lambda = 0$ gives the following conditions:

$$\begin{cases} \lambda_1 w^2 = -\frac{A}{T^3} \\ w^2 = \frac{2B-A+1}{T^2} \\ \lambda_1 = \frac{A-B-2}{T} \end{cases} \quad (11)$$

and therefore, for a bifurcation to occur A must be above a threshold A^c, defined as:

$$A^c = \frac{4+3B-\sqrt{8+4B+B^2}}{2} \quad (12)$$

a monotonically increasing function of B.

And at the bifurcation threshold, the period τ deduced from the linear stability analysis is defined as:

$$\tau^2 = 1/w^2 = \frac{2T^2}{-2+B+\sqrt{8+4B+B^2}} \quad (13)$$

a monotonically decreasing function of B. As expected, the period scales with the turnover rate, which is the only timescale of the problem here.

As a consistency check, one can notice that in the system 11, the condition $w^2 > 0$ (the imaginary parts of the second and third eigenvalues is non-zero), leads to $\lambda_1 < 0$ for any $A > 0$.

This indicates that the first eigenvalue is indeed strictly negative here, and does not take part in the instability.

Interestingly, we thus see that for identical turnover time of all three species, the negative interaction between MBS and MyoII is not required for oscillations: $B = 0$ still leads to oscillations, due to the interplay between the positive interaction of Rock on MyoII, positive regulation of MyoII on MBS and the negative interaction of MBS on ROCK. On the other hand, the positive interaction of ROCK on MyoII is crucial for the oscillations, as the condition $A = 0$ does not allow for oscillations.

1.6 Three species general cases

Using the previous methods on the generic three-species system, we can calculate that again, a bifurcation to a stationary solution occurs for A above a threshold A^c, defined as:

$$2A^c = 2+3B+\tau_m(1/\tau_R+1/\tau_M) - \sqrt{4(1+2B)\tau_m/\tau_R+(\tau_m(1/\tau_R+1/\tau_M)-B)} \quad (14)$$

which is still a monotonically increasing function of B. For $B = 0$, both the threshold monotonically decreases with both τ_m and τ_M. And, at the bifurcation threshold, the period τ deduced from the linear stability analysis is defined as:

$$\tau^2 = \frac{2\tau_M^2\tau_m\tau_R}{B\tau_M\tau_R - \tau_m\tau_R - \tau_m\tau_M + \sqrt{4(1+2B)\tau_M^2\tau_m\tau_R + (\tau_m\tau_M+\tau_m\tau_R - B\tau_M\tau_R)^2}} \quad (15)$$

Importantly, we verified these analytical criteria by calculating numerically a phase diagram in (A,B) space (Supplementary Fig. 6a-d). We systematically checked for the signsand real/imaginaryparts of the eigenvalues, and found that the only instability occurred asa pair of conjugate eigenvalues had a positive real parts (in particular, we didn't find a region ofthe phase diagramin which a single real eigenvalues became positive, which wouldindicate a loss of stability of the homogeneous solution without oscillations). We alsosystematically checked that the numerical solutions always remained strictly positive, and plotted three examples of numericalsolutions (indicated as x, y and z) in three different regionsof the phase diagram, for illustration (Supplementary Fig. 6e).

2 Fitting procedure

In this section, we describe the fitting procedure used to define the parameters involved in the model described above.

2.1 Determining characteristic turnover times

In principle, one could use the oscillation curves from Rock, MyoII and MBS to fit all of the aforementioned parameters. In practise, the number of parameters is too large and leads to very high indeterminacy on each parameter value. In order to put additional constraints on the model, we thus performed Flurorescence Recovery After Photobleaching (FRAP) assays, in order to extract the three characteristic turnover times τ_T, τ_m and τ_M involved in the model. We successively performed FRAP assays on the basal central pool of Rok-GFP, MyoII-GFP and MBS-GFP. Interestingly, we found that recovery were consistently well-fitted by single-exponentials, which validates *a posteriori* our assumptions above of taking first-order kinetics (see Materials and methods for details).

From samples taken at stage LS9 (Fig. 5c and Supplementary Fig. 5), we could measurecharacteristic times of recovery (different from the half-time showed in main textby a factor
$ln(2)$) of $\tau_m = 86 \pm 43s$, $\tau_R = 21 \pm 10s$ and $\tau_{MBS} = 9 \pm 6s$. Interestingly, this very short time scale for the recovery of MBS indicated that although it has a key role in the downregulation of MyoII intensity, its dynamics cannot the limiting one in this system, as otherwise, one would predict that MBS should follow MyoII extremely closely (i.e. on order of $5s$ instead of around a minute). This lead us to investigate whether other members of the MyoII phosphatase machinery had longer dynamics, and concentrated of Flw, given its previously reported importance in oocyte oscillations[13], and has we found that its oscillation trails MyoII by the same delay as MBS (Supplementary Fig. 2). Importantly, we repeated the same sets of FRAP experiments on Flw, and found $\tau_{flw} = 54 \pm 21s$ (Fig. 5c and Supplementary Fig. 5), compatible with the lag time between MyoII and MBS/flw, and arguing that its dynamics is the limiting one to drive MyoII dephosphorylation. We thus use these parameters as inputs to the model.

Once these turnover parameters have been fixed, only two rescaled parameters are left in the problem, i.e. the interaction coefficients B and A, quantifying respectively the effect of MBS and ROCK on MyoII concentration. As the model that we describe throughout this Supplementary Text has no closed-form analytical solutions in the general case, we resort to numerical integration of the corresponding equations, for various values of B and A

In each case described below, we validate from a coarse parameter sweep the analytical criteria from Eq. (14-15) to the transition threshold for oscillations, with oscillations occurring above a critical value for A which depends in a quasi-affine fashion upon the value of B. One should also note that due to the values of the characteristic time, the model also reproduces accurately the relative amplitudes of the three species: largest amplitude for ROCK (as it has more time to build up due to its low τ_R), and smallest amplitude for MBS/Flw oscillation. Interestingly, we also found that the predicted period for the oscillation is rather insensitive to these parameters close to the transition, and matches well the observed oscillation time of around $7min$ for wild-type, in a robust way close to the transition point, so that the exact value of A and B close to the transition is largely irrelevant. For the graphs of Fig. 5, we used the parameters $A = 0.8$ and $B = 0.5$.

3 Perturbation experiments, and relationship between the period and amplitude of the oscillations

Next, we investigated how changes in the interaction between ROCK, MyoII, and MBS/flw would change theoretically the period and amplitude of the MyoII oscillation. As mentioned above, the generic system of equation describing our oscillator is

$$\begin{cases} \frac{dR}{dt} = \frac{R}{\tau_R}(1-M) \\ \frac{dm}{dt} = -\frac{m}{\tau_m}(1+BM-AR)+\frac{J}{\tau_m} \\ \frac{dM}{dt} = \frac{m-M}{\tau_M} \end{cases} \tag{16}$$

once all of the average concentrations have been normalized to 1 (so that $J = 1+B-A$ for the control parameters).

However, in the case of perturbation experiments, these average concentrations will be affected, which needs to be accounted for. We first start by discussing the effect of ROCK activity modulation.

3.1 ROCK activity modulation

As shown in Fig. 6i-p, constitutively active and dominant negative ROCK modulate the levels of both MyoII and ROCK itself.

A simple way to take this into account is to consider a modulation in the self-activation feedback of ROCK, defining R_0 as:

$$\begin{cases} \frac{dR}{dt} = \frac{R}{\tau_R}(R_0 - M) \\ \frac{dm}{dt} = -\frac{m}{\tau_m}(1 + BM - AR) + \frac{J}{\tau_m} \\ \frac{dM}{dt} = \frac{m-M}{\tau_M} \end{cases} \tag{17}$$

Thus, constitutively active (resp. dominant negative) versions of ROCK correspond to large (resp. small) values of R_0. Importantly, we then assessed how variations of R_0 modified both the amplitude of the oscillation (assessed via the standard deviation of the concentration in time), but also its period. We first checked how the amplitude of the oscillation evolved, and found, as expected, that below a threshold, no oscillations occur (which would correspond to dominant negative Rho). We then observe a continuous phase transition, with amplitudes increasing above the threshold. Fig. 5 shows three examples for $R_0 = 0.925$ (Fig. 5f), $R_0 = 1$ (Fig. 5d), and $R_0 = 1.2$ (Fig. 5e), while Supplementary Fig. 6e shows the general phase diagram as a function of R_0.

Moreover, when plotting the amplitude vs period of the oscillation, while varying R_0, we noticed a robust and positive relationship between the two (Fig. 5g), which was insensitive to the position within the (A,B) phase diagram. This strongly echoes the experimental observation, where constitutive activation of ROCK increases both amplitude and period, while dominant negative versions of ROCK did the reverse. It should be noted that, as observed experimentally, and although we mainly report amplitude vs period relationships here, activation (resp. inhibition) of ROCK results also in a global increase (resp. decrease) of the time-averaged MyoII concentration.

Finally, and importantly, it should be noted that this positive correlation between amplitude and period held under a different assumption on the exact nature of ROCK activation. Indeed, one could also conceive that ROCK constitutive activation does not increase the self-activation of ROCK per se, but rather makes ROCK less sensitive to MBS/flw driven inhibition, modelled as a coefficient R_1, such as:

$$\begin{cases} \frac{dR}{dt} = \frac{R}{\tau_R}(1 - M/R_1) \\ \frac{dm}{dt} = -\frac{m}{\tau_m}(1 + BM - AR) + \frac{J}{\tau_m} \\ \frac{dM}{dt} = \frac{m-M}{\tau_M} \end{cases} \tag{18}$$

Importantly, when performing the same analysis as above, we found a similar positive relationship between period and oscillation.

3.2 MBS activity modulation

As shown in Fig. 6q-x, constitutively active and dominant negative MBS also modulate thelevels of both MyoII and ROCK.

In close similarity to our treatment of ROCK above, a simple way to take this into account is to consider a modulation in the MyoII-driven recruitment strength of MBS/flw, defining M_0 as:

$$\begin{cases} \frac{dR}{dt} = \frac{R}{\tau_R}(1 - M) \\ \frac{dm}{dt} = -\frac{m}{\tau_m}(1 + BM - AR) + \frac{J}{\tau_m} \\ \frac{dM}{dt} = \frac{mM_0-M}{\tau_M} \end{cases} \tag{19}$$

Thus, constitutively active (resp. dominant negative) versions of MBS correspond to large (resp. small) values of M_0. A first natural consequence of increasing M_0 is that although it does not change the steady-state value of M, it decreases both the steady-state concentration of MyoII (i.e. to $1/M_0$), as expected (Fig. 5g), but also, and as observed experimentally (Fig. 5a), the steady-state concentration of ROCK.

Importantly, we then assessed how variations of M_0 modified the amplitude and period of the oscillation. We again noticed a robust and positive relationship between the two (Fig. 5g), which matched very closely the one observed for ROCK activity modulation. This strongly echoes the experimental observation, where constitutive activation of MBS decreases both amplitude and period, while dominant negative versions of MBS did the reverse.

Therefore, both the analysis of ROCK and MBS activity modulation predict a robust positive relationship between average concentration, oscillation period, and oscillation amplitude, which we observe experimentally. Further work would be needed to understand in better detail how exactly each genetic perturbation modifies each coefficient of the model quantitatively.

4 Spatial organisation of the chemical oscillator

As discussed in the main text, an additional feature of the data is the spatial dependency of the oscillation. In the previous sections, we have assumed that all concentrations are homogeneous in space, and depend only on time. This is equivalent to assuming that diffusion is sufficiently large to homogenize spatially all concentrations. However, we have seen in the main text that ROCK is typically activated at the cellular junctions first, which is the location of its main activator Rho, before quickly reaching the center of the cell in a wave pattern. In order to account theoretically for this observed fact, we need to re-write the previous set of ordinary differential equation into a set of partial differential equations. The reaction-diffusion equations associated with ROCK, MyoII and MBS dynamics is then:

$$\begin{cases} \frac{\partial R}{\partial t} = D_R \Delta R + \frac{R}{\tau_R}(1-M) \\ \frac{\partial m}{\partial t} = D_m \Delta m - \frac{m}{\tau_m}(1+BM-AR) + \frac{J_0}{\tau_m} \\ \frac{\partial M}{\partial t} = D_M + \Delta M \frac{m-M}{\tau_M} \end{cases} \tag{20}$$

with D_R, D_m and D_M the two-dimensional diffusion coefficients of respectively ROCK, MyoII, and MBS/flw, and Δ the Laplace operator. These equations are complemented with no-flux boundary conditions, $\partial_n m = \partial_n M = 0$, expressing the fact that there is no flux of ROCK, MyoII or MBS/flw through the cell junction (**n** being the local normal vector to the boundary of the integration domain).

In the case of such spatially uniform coefficients, we get uniform oscillations in the cell for realistic values of the diffusion coefficients, in a manner qualitatively equivalent to the zero-dimension case. Inspired by the FRAP experiments, we set the coefficient of diffusion of MyoII to zero, given its low 2D diffusion properties.

4.1 1D simulation

However, and as discussed in the main text, this is clearly unrealistic: ROCK activation can only occur at the cell boundary, as this is where its upstream activator, Rho, is located. This is consistent with the fact that we observe ROCK amplification occurring at first at the cell boundary. Therefore, in a cell however, symmetry is broken by the presence of Rho at the boundary, locally activating ROCK, and we add in our model this feature, by modelling it as a source of ROCK in the proximity of the boundaries, i.e. on a characteristic distance x_b very

small compared to the typical cell length L (we take $x_b = 0.02L$ in the simulations). In one dimension, this reads:

$$\begin{cases} \frac{\partial R}{\partial t} = D_R \partial_{xx} R + \frac{R}{\tau_R}(1-M) + \frac{\alpha_R}{\tau_R} H(x_b - x) \\ \frac{\partial m}{\partial t} = D_m \partial_{xx} m - \frac{m}{\tau_m}(1 + BM - AR) + \frac{J_0}{\tau_m} \\ \frac{\partial M}{\partial t} = D_M \partial_{xx} M \frac{m-M}{\tau_M} \end{cases} \tag{21}$$

with $H(x)$ the Heaviside step function, equal to zero in the cell bulk, and equal to one close to the $x = 0$ boundary, and α_R a parameter which quantifies the relative importance of the Rho source compared ROCK auto-activation (schematics on Fig. 5h). Varying α_R reveals that it does not have a strong effect on the resulting qualitative dynamics, as long as it is not negligible. Strikingly, simulating the model dynamics revealed waves of ROCK/MyoII propagating from the boundary, reproducing the experimental data (see Fig. 5i,j). In the kymographs of Fig. 5i,j (generated using the NDSolve function in Mathematica), we used $\alpha_R = 0.2$, together with $D_m = 0$, $D_M = 0.01$ and $D_R = 0.025$. As expected, one can increase (resp. decrease) the velocity of the flow by increasing (resp. decreasing) the diffusion coefficient of ROCK D_r. In future studies, it would be interesting to calculate how this velocity is selected, as well as compare it to the data, although the complex spatial structure of the basal surface of the cell (i.e. for instance the fact that MyoII is less dense near the boundaries) could complicate the comparison.

4.2 2D simulation

Moreover, to verify the robustness of this model prediction, and in particular to check the stability of this wave propagation in two-dimension, we performed the same simulations on a disk, with preferential activation of ROCK at the edge of the disk $H(r-L-x_b)$. We performed these simulations with a Finite Element Method (using FreeFem++), and the results are reported on Supplementary Fig. 6i-k: in particular, we observed no qualitative difference with the 1D predictions,with temporal oscillations and inward wave propagation. ROCK/MyoII diffusion waves propa-gated towards the center of the disk, until they were "caught" up by MBS/flw, causing the peakto dis-assemble (Supplementary Fig. 6j). We also performed two-dimension simulationsin the case where ROCK activation occurred preferentially at one side of the disk only, tomimic our experimental observation that ROCK activation starts preferentially on one side ofa cell (Supplementary Fig. 6i). Further work would be neededto understand the origin ofsuch a symmetry breaking. However, we again observe abehavior qualitatively similar to the1D simulation, with directed propagation of pulses.

In conclusion, this suggests that MyoII oscillation at the basal cortex of the epithelial follicle cells of the *Drosophila* oocyte can be understood as a biochemical oscillator involving the key regulators of MyoII (ROCK and MBS/flw). Further work will need to address whether the positive and negative interactions that make up the core of our model are direct, or result from more complex interaction networks.

Supplementary References

1. Munjal, A., Philippe, J.M., Munro, E. & Lecuit, T. A self-organized biomechanical network drives shape changes during tissue morphogenesis. *Nature* **524**, 351-355 (2015).
2. Phair, R.D., Gorski, S.A. & Misteli, T. Measurement of dynamic protein binding to chromatin in vivo, using photobleaching microscopy. *Methods Enzymol* **375**, 393-414 (2004).
3. Ellenberg, J., Lippincott-Schwartz, J. & Presley, J.F. Two-color green fluorescent protein time-lapse imaging. *Biotechniques* **25**, 838-842, 844-836 (1998).
4. Novak, B. & Tyson, J.J. Design principles of biochemical oscillators. *Nat Rev Mol Cell Biol* **9**, 981-991 (2008).
5. Riento, K. & Ridley, A.J. Rocks: multifunctional kinases in cell behaviour. *Nat Rev Mol Cell Biol* **4**, 446-456 (2003).
6. Wilkinson, S., Paterson, H.F. & Marshall, C.J. Cdc42-MRCK and Rho-ROCK signalling cooperate in myosin phosphorylation and cell invasion. *Nat Cell Biol* **7**, 255-261 (2005).
7. Scholey, J.M., Taylor, K.A. & Kendrick-Jones, J. Regulation of non-muscle myosin assembly by calmodulin-dependent light chain kinase. *Nature* **287**, 233-235 (1980).
8. Kimura, K. *et al.* Regulation of myosin phosphatase by Rho and Rho-associated kinase (Rho-kinase). *Science* **273**, 245-248 (1996).
9. Ito, M., Nakano, T., Erdodi, F. & Hartshorne, D.J. Myosin phosphatase: structure, regulation and function. *Mol Cell Biochem* **259**, 197-209 (2004).
10. Alessi, D., MacDougall, L.K., Sola, M.M., Ikebe, M. & Cohen, P. The control of protein phosphatase-1 by targetting subunits. The major myosin phosphatase in avian smooth muscle is a novel form of protein phosphatase-1. *Eur J Biochem* **210**, 1023-1035 (1992).
11. Totsukawa, G. *et al.* Activation of myosin phosphatase targeting subunit by mitosis-specific phosphorylation. *J Cell Biol* **144**, 735-744 (1999).
12. Lee, A. & Treisman, J.E. Excessive Myosin activity in mbs mutants causes photoreceptor movement out of the Drosophila eye disc epithelium. *Mol Biol Cell* **15**, 3285-3295 (2004).
13. Valencia-Exposito, A., Grosheva, I., Miguez, D.G., Gonzalez-Reyes, A. & Martin-Bermudo, M.D. Myosin light-chain phosphatase regulates basal actomyosin oscillations during morphogenesis. *Nat Commun* **7**, 10746 (2016).
14. Chen, X.Q. *et al.* Characterization of RhoA-binding kinase ROKalpha implication of the pleckstrin homology domain in ROKalpha function using region-specific antibodies. *J Biol Chem* **277**, 12680-12688 (2002).
15. Totsukawa, G. *et al.* Distinct roles of ROCK (Rho-kinase) and MLCK in spatial regulation of MLC phosphorylation for assembly of stress fibers and focal adhesions in 3T3 fibroblasts. *J Cell Biol* **150**, 797-806 (2000).
16. Kawano, Y. *et al.* Phosphorylation of myosin-binding subunit (MBS) of myosin phosphatase by Rho-kinase in vivo. *J Cell Biol* **147**, 1023-1038 (1999).
17. Wang, Y. *et al.* ROCK isoform regulation of myosin phosphatase and contractility in vascular smooth muscle cells. *Circ Res* **104**, 531-540 (2009).
18. Winter, C.G. *et al.* Drosophila Rho-associated kinase (Drok) links Frizzled-mediated planar cell polarity signaling to the actin cytoskeleton. *Cell* **105**, 81-91 (2001).
19. Turing, A.M. The Chemical Basis of Morphogenesis. *Philosophical Transactions of the Royal Society of London. Series B, Biological Sciences* **237**, 37-72 (1952).

20. FitzHugh, R. *FitzHugh, R.: Mathematical models of threshold phenomena in the nerve membrane. Bull. Math. Biophys* ***17****, 257-278* (1955).

21. Bomze, I.M. Lotka-Volterra equation and replicator dynamics: a two-dimensional classification. *Biol. Cybernetics* ***48***, 201-211 (1983).
