# Supplementary material for: A biochemical network controlling basal myosin oscillation
Source: Nat Commun. 2018 Mar 23;9:1210. doi: 10.1038/s41467-018-03574-5 (PMC5865161; doi:10.1038/s41467-018-03574-5)
Supplement: Supplementary file 3 — Description of Additional Supplementary Files(PDF 176 kb) [file 41467_2018_3574_MOESM3_ESM.pdf]

Description of Additional Supplementary Files

File Name: Supplementary Movie 1
Description: Time-lapse series of the representative wild type follicle cell, labelled with Rho1-GFP and MyoII-mCherry. Time interval is 1 min, and scale bar is 5 µm.

File Name: Supplementary Movie 2
Description: Time-lapse series of the representative wild type follicle cell, labelled with ROCK-GFP and MyoII-mCherry. Time interval is 1 min, and scale bar is 5 µm.

File Name: Supplementary Movie 3
Description: Time-lapse series of the representative wild type follicle cell, labelled with MBS-GFP and MyoII-mCherry. Time interval is 0.5 min, and scale bar is 5 µm.

File Name: Supplementary Movie 4
Description: Time-lapse series of the representative wild type follicle cell, labelled with Flw-YFP and MBS-RFP. Time interval is 0.5 min, and scale bar is 5 µm.

File Name: Supplementary Movie 5
Description: Time-lapse series of the representative wild type follicle cell, labelled with Flw-YFP and MyoII-mCherry. Time interval is 0.5 min, and scale bar is 5 µm.

File Name: Supplementary Movie 6
Description: Time-lapse series of the representative wild type follicle cell, labelled with ROCK-GFP and Rho1-RFP. Time interval is 1 min, and scale bar is 5 µm.

File Name: Supplementary Movie 7
Description: Time-lapse series of the representative wild type follicle cell, labelled with ROCK-GFP and MyoII-mCherry, and illuminated with blue light. Time interval is 30 sec, and scale bar is 5 µm.

File Name: Supplementary Movie 8
Description: Time-lapse series of the representative LARIAT-expressing follicle cell, labelled with ROCK-GFP and MyoII-mCherry, and illuminated with blue light. Time interval is 30 sec, and scale bar is 5 µm.

File Name: Supplementary Movie 9
Description: Time-lapse series of the representative wild type follicle cell, labelled with Flw-YFP and MyoII-mCherry, and illuminated with blue light. Time interval is 30 sec, and scale bar is 5 µm.

File Name: Supplementary Movie 10
Description: Time-lapse series of the representative LARIAT-expressing follicle cell, labelled with Flw-YFP and MyoII-mCherry, and illuminated with blue light. Time interval is 30 sec, and scale bar is 5 µm.

File Name: Supplementary Movie 11
Description: Time-lapse series of the representative wild type follicle cell, labelled with MBS-GFP and MyoII-mCherry, and illuminated with blue light. Time interval is 30 sec, and scale bar is 5 µm.

File Name: Supplementary Movie 12
Description: Time-lapse series of the representative LARIAT-expressing follicle cell, labelled with MBS-GFP and MyoII-mCherry, and illuminated with blue light. Time interval is 30 sec, and scale bar is 5 µm.
